# Structural degradation of tungsten sandwiched in hafnia layers determined by in-situ XRD up to 1520 °C

**DOI:** 10.1038/s41598-021-82821-0

**Published:** 2021-02-08

**Authors:** Gnanavel Vaidhyanathan Krishnamurthy, Manohar Chirumamilla, Surya Snata Rout, Kaline P. Furlan, Tobias Krekeler, Martin Ritter, Hans-Werner Becker, Alexander Yu Petrov, Manfred Eich, Michael Störmer

**Affiliations:** 1 Institute of Materials Research, Helmholtz-Zentrum Geesthacht, Max-Planck-Strasse 1, 21502 Geesthacht, Germany; 2 Institute of Optical and Electronic Materials, Hamburg University of Technology, Eissendorfer Strasse 38, 21073 Hamburg, Germany; 3 Electron Microscopy Unit, Hamburg University of Technology, Eissendorfer Strasse 42, 21073 Hamburg, Germany; 4 Institute of Advanced Ceramics, Hamburg University of Technology (TUHH), Denickestraße 15, 21073 Hamburg, Germany; 5 Central Unit for Ionbeams and Radionuclides, Ruhr-Universität Bochum, Universitätstraße 150, 44801 Bochum, Germany; 6 ITMO University, 49 Kronverkskii Avenue, Saint Petersburg, 197101 Russia

**Keywords:** Metamaterials, Characterization and analytical techniques, Materials science, Optics and photonics

## Abstract

The high-temperature stability of thermal emitters is one of the critical properties of thermophotovoltaic (TPV) systems to obtain high radiative power and conversion efficiencies. W and HfO_2_ are ideal due to their high melting points and low vapor pressures. At high temperatures and given vacuum conditions, W is prone to oxidation resulting in instantaneous sublimation of volatile W oxides. Herein, we present a detailed in-situ XRD analysis of the morphological changes of a 3-layer-system: HfO_2_/W/HfO_2_ layers, in a high-temperature environment, up to 1520 °C. These samples were annealed between 300 °C and 1520 °C for 6 h, 20 h, and 40 h at a vacuum pressure below 3 × 10^–6^ mbar using an in-situ high-temperature X-ray diffractometer, which allows investigation of crucial alterations in HfO_2_ and W layers. HfO_2_ exhibits polymorphic behavior, phase transformations and anisotropy of thermal expansion leads to formation of voids above 800 °C. These voids serve as transport channels for the residual O_2_ present in the annealing chamber to access W, react with it and form volatile tungsten oxides. An activation energy of 1.2 eV is calculated. This study clarifies the limits for the operation of W-HfO_2_ spectrally selective emitters for TPV in high-temperature applications.

## Introduction

A major portion of industries globally, still rely on fossil fuels as their primary resource of energy^1^. A significant amount of this energy is wasted as unexploited heat, named waste heat. One means of efficiently utilizing the wasted heat to harness electricity can be realized by using a thermophotovoltaic (TPV) system^2^. A TPV system can as well produce electric power from fossil fuels^3^, solar radiation^4–8^ and in radioisotope thermoelectric generators (RITEGs)^9–11^. In TPV, an emitter is heated to high-temperatures, typically above 1000 °C, and its radiant thermal energy is directly converted into electricity using photovoltaic (PV) cells^12–15^. The efficiency of the TPV system can be enhanced by using spectrally selective emitters^16–23^. Such selective emitters are designed to precisely emit spectrally tailored radiation that matches the band gap of the PV cell. One of the critical parameters for obtaining high power and efficiency is operating the emitters at high-temperature, as the radiated power^24^ of a black body and also of a selective thermal emitter is proportional to *T*^4^ according to the Stefan Boltzmann law^25^ and the spectral maximum shift to shorter wavelengths proportional to 1/*T* which is known as Wien’s displacement law and can be derived from Planck’s law of radiation. This implies that the emitter should work at 1400 °C temperature, to match the bandgap *E*_*g*_ of high efficiency GaSb PV cell at 0.72 eV^26^. It is worth mentioning that besides increasing the total radiated power by almost a factor of 4, a raise in temperature from 1000 to 1400 °C will also increase the TPV efficiency of a metamaterial emitter from 33 to 48%^27^. Therefore, high-temperature stability and long lifetime at these temperatures are required for the effective performance of a TPV system.

In this perspective, appealing choices of metals with high melting point are Mo (2620 °C), Ta (3020 °C) and W (3410 °C), while Al_2_O_3_ (2054 °C), ZrO_2_ (2715 °C), and HfO_2_ (2800 °C) are suitable alternatives among the dielectrics^28–30^. Photonic crystals^31^, gratings^32^, and multilayered metamaterials^33^ comprising of alternate layers of metal and dielectric are examples of selective emitters. Arpin et al.^34^ demonstrated a stability of 1000 °C in their W based 3D photonic crystal prepared by conformal deposition of W using atomic layer deposition (ALD). Yokoyama et al.^35^ used a combination of Mo and Al_2_O_3_ to fabricate a metal–insulator–metal thermal emitter that reached an operational temperature of 1000 °C. Thermal stability of a W/Al_2_O_3_ based metasurface solar absorber up to 1200 °C was demonstrated by Chang et al.^36^ using in-situ high-temperature reflectance experiments. Kim et al.^37^ reported a multilayer metamaterial design consisting of W/Al_2_O_3_ layers, which showed thermal stability up to 1200 °C for 3 h. The degradation in optical properties was observed due to the agglomeration of W within the layers with further increase in the temperature. Kohiyama et al.^38^ and Shimizu et al.^39^ carried out high-temperature experiments up to 1360 °C on selective absorber/emitters, which were a combination of layered multilayers of Mo/HfO_2_ and W/Yttria stabilized zirconia (YSZ). Often the cause of structural failure at high-temperatures is due to oxidation^33,40–42^ and surface diffusion^43–45^ in the layered stacks. The other notable mechanisms that are observed at high-temperatures are stress induced in the multilayers leading to delamination and cracking^46^.

In a more general perspective for high-temperature stable metamaterials, not only the melting point but as well the vapor pressure^47^ of both layer materials is important for a good thermal stability. It is known that materials which attain a high vapor pressure below their melting point on heating, tend to sublimate directly into the gaseous phase at a temperature below their melting point^48^. Therefore, the materials selected for a high-temperature application should possess a low vapor pressure: W and HfO_2_ are exceptional in their classes of materials. The vapor pressure of W is 8.15 × 10^–10^ mbar at 2000 °C^49^ is the lowest among metals and that of HfO_2_ is 1 × 10^–13^ mbar at 1732 °C^50^, shows exceptional low vapor pressure among dielectrics.

**Figure 1:**
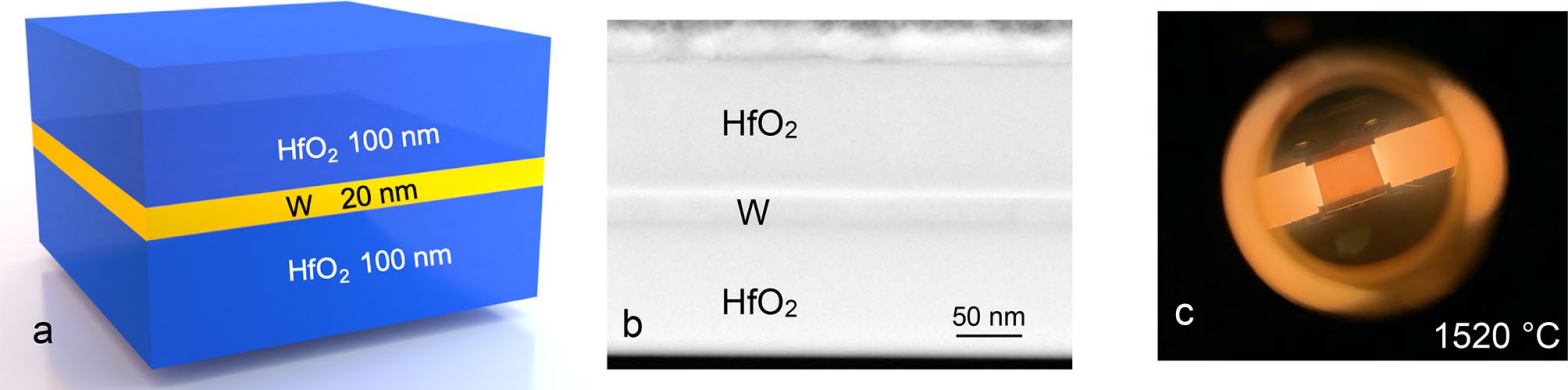
(**a**) Schematic of the 3-layer-system used in the annealing experiment (**b**) scanning transmission electron microscope (STEM) image of an as-prepared 3-layer-system; prior to focused ion beam (FIB) milling a thin layer of C was deposited above the top HfO_2_ layer to circumvent charging of the substrate (the sapphire substrate is not shown) (**c**) Image of the 3-layer-system at 1520 °C inside the heating chamber.

Herein, we focus on in-situ X-ray diffraction (XRD) experiments of a simplified 3-layer-system of 100 nm HfO_2_/20 nm W/100 nm HfO_2_ shown (Fig. 1a–c) in order to investigate time-dependence of the structural and morphological changes of the layer materials at high-temperatures. The repetition of a 3-layer-system can be used to construct a multilayered selective emitter. The 3-layer-system is prepared by magnetron sputtering, a method well established for depositing refractory materials, that facilitates the growth of highly uniform and precise multilayers^51–53^. The primary goal of using a 3-layer-system is to understand the mechanism of oxidation in a single W layer that leads to initial structural changes, followed by degradation at various annealing temperatures and durations. Chemical composition and morphology of the 3-layer-systems after preparation and after different annealing experiments were characterized by scanning transmission electron microscopy—energy dispersive X-ray spectroscopy (STEM-EDS). Further, we discuss the changes observed in the 3-layer-system due to high operational temperatures such as phase formation, changes in interplanar spacings and grain sizes, and alterations of the volume fractions of the observed phases. Structural degradation mechanisms of W films at high vacuum conditions and above 1000 °C operational temperatures are demonstrated, which reveals the current limitations in the life-time of W-based metamaterials. These measurements allow a better understanding of the underlying mechanisms of the failure of W/HfO_2_-multilayered metamaterials at high-temperatures.

## Results and discussion

**Figure 2:**
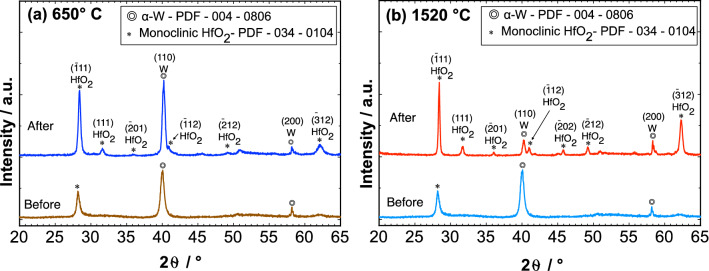
Diffraction patterns of the two identical 3-layer-systems: 100 nm HfO_2_/20 nm W/100 nm HfO_2_, measured before and after annealing at 650 °C (**a**) and 1520 °C (**b**) for 6 h.

Ex-situ X-ray diffraction measurements of two identical 3-layer-systems after 6 h annealing experiments allow us to investigate the changes of crystal structure and phase formation under high vacuum conditions at two different temperatures. Slight variations are noticeable after the moderate annealing at 650 °C (Fig. 2a) and distinct changes are visible after 1520 °C (Fig. 2b). Before annealing, it is always important to control that the as-prepared samples are comparable, which can be confirmed by nearly identical diffraction patterns, which contain only a few peaks that corresponds to three phases in the recorded range. The peaks (110) at 39.9° and (200) at 58.3° correspond to the body centered cubic (bcc) phase of α-W. The α-phase is the thermodynamically stable phase of W and the metastable ß-phase of W is not present in our films^54^. In the case of HfO_2_, a coexistence of a nanocrystalline monoclinic phase and an amorphous phase is observed after preparation. The (− 111) peaks at 28.3° can be attributed to the monoclinic phase of HfO_2_, whereas the broad bump around 30.4° corresponds to the amorphous phase of HfO_2_. In the case of the monoclinic HfO_2_, the full width at half maximum of the (− 111) peak is only 0.48°, whereas the amorphous phase of HfO_2_ at 30.4° possesses a broad peak with a FWHM value of 5.2°. From the peak shape of the diffraction pattern, it is estimated (using Bruker EVA software) that the as-prepared HfO_2_ layers have volume fraction of 56% and 44% for the crystalline and amorphous phase after preparation. Due to annealing, already after 650 °C, the crystallinity of HfO_2_ is increased, since the broad peak of the amorphous phase has vanished, and some new crystalline peaks of the monoclinic phase appeared. Furthermore, after 1520 °C, the peaks become sharper and a number of new peaks are identified, all of them attributed to the monoclinic phase of HfO_2_. A slight change in the (110) W peak position to 40.1° is observed, since the bcc W structure relaxes towards the thermodynamic equilibrium position of bulk W, which is 40.265° (PDF card 00-004-0806) at room temperature. These changes are discussed later in more detail. It is most likely that new peaks from the monoclinic phase of HfO_2_ occur during annealing as the existing grains grow^55^, in order to attain a new position due to relaxation. In summary, after annealing to either 650 °C or 1520 °C only two phases are evident, the bcc phase from W and the monoclinic phase from HfO_2_. It is worthwhile to mention that there is no evidence of any solid-state reactions between the two-layer materials in the 3-layer-system such as WO_x_, Hf-W-O alloys, and pure Hf. The two obtained phases only present small shifts in the peak positions for two different annealing temperatures, but there is no hint for any phase or composition change during annealing.

In an in-situ annealing experiment, one can clearly visualize transient phase formation and their changes as a function of time and temperature. Diffraction patterns were recorded sequentially during ramp-up of the temperature. Using controlled heating rates enables us to pursue reaction kinetics and material transport processes undergone by the sample.

**Figure 3:**
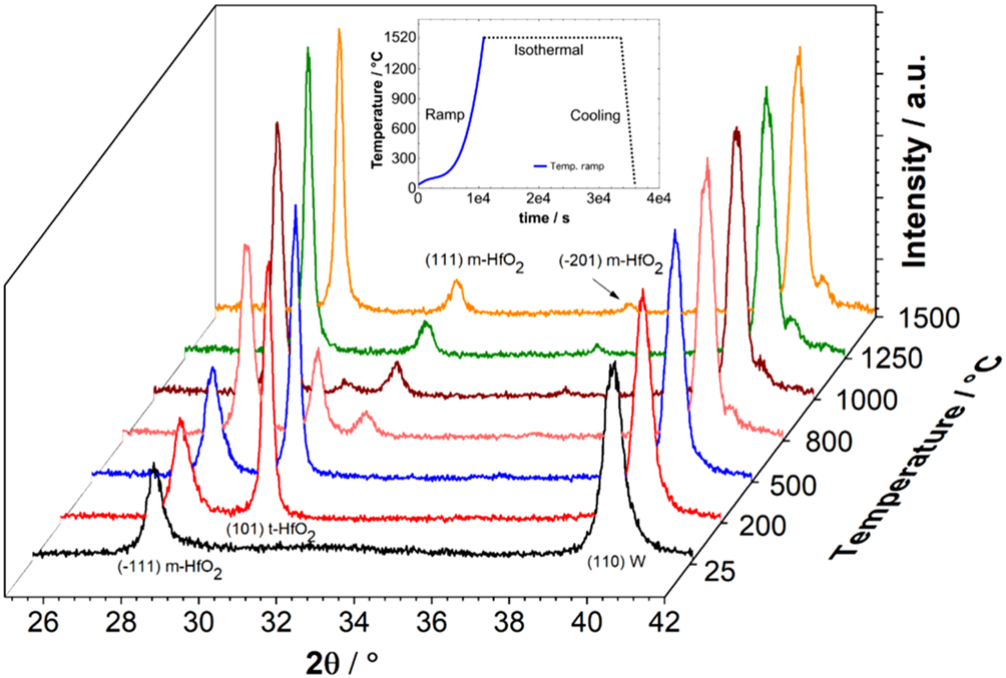
In-situ XRD 2θ scans of a 3-layer-system from room temperature until 1520 °C. During this temperature increase (represented by thick blue line in the inset, which shows the whole annealing experiment) a tetragonal phase of HfO_2_ appears around 200 °C and later slowly disappears above 800 °C.

The diffraction patterns of a 3-layer-system recorded at different temperatures during the ramp part of the annealing experiments are shown in Fig. 3, from room temperature up to 1520 °C. In the first instance, it is clearly noticeable that the peaks get sharper as the temperature increases and move to higher angles due to relaxation approaching the equilibrium value at the adjusted temperature. The broad, amorphous bump of HfO_2_ at room temperature slowly starts to vanish. It is well known from the literature that HfO_2_ exhibits polymorphic behavior^30^. A new peak (101) from the tetragonal phase of HfO_2_ (Supplementary Figs. 1, 2) emerges from the amorphous region at 30.3°, as the temperature rises to 200 °C. This peak continues to grow in intensity and tends to become narrower until the ramp temperature reaches 750 °C, after which there is a decline in (101) peak intensity. The d-values of this tetragonal phase are nearly constant with *d*_101_ = 0.2951 nm at 200 °C and *d*_101_ = 0.2968 nm at 750 °C, and this small change is associated to thermal expansion. On the other hand, the intensity of the (− 111) peak, the strongest X-ray spot from the monoclinic HfO_2_ phase starts to grow higher in intensity, where it is doubled at 500 °C and tripled at 1250 °C. This is a clear indication that the tetragonal phase formed at 200 °C is transformed into the highly stable monoclinic phase at high temperatures. These results are in good agreement to the experiments carried out by Ushakov et al.^56^ and Zhao et al.^57^, as they observed amorphous HfO_2_ phases that crystallize into tetragonal phase first and later transform into the stable monoclinic phase in thin films, which were prepared by sol–gel, pulsed laser deposition (PLD)^56^ and atomic layer chemical vapor deposition (ALCVD)^57^. As the temperature increases above 1000 °C, we do not see any evidence for a tetragonal phase, but more new peaks corresponding to the monoclinic phase of HfO_2_ start to appear such as (111) and (− 201). We do not observe any phase change in the W layer except the sharpening of the (110) W peak during the heating ramp.

**Figure 4:**
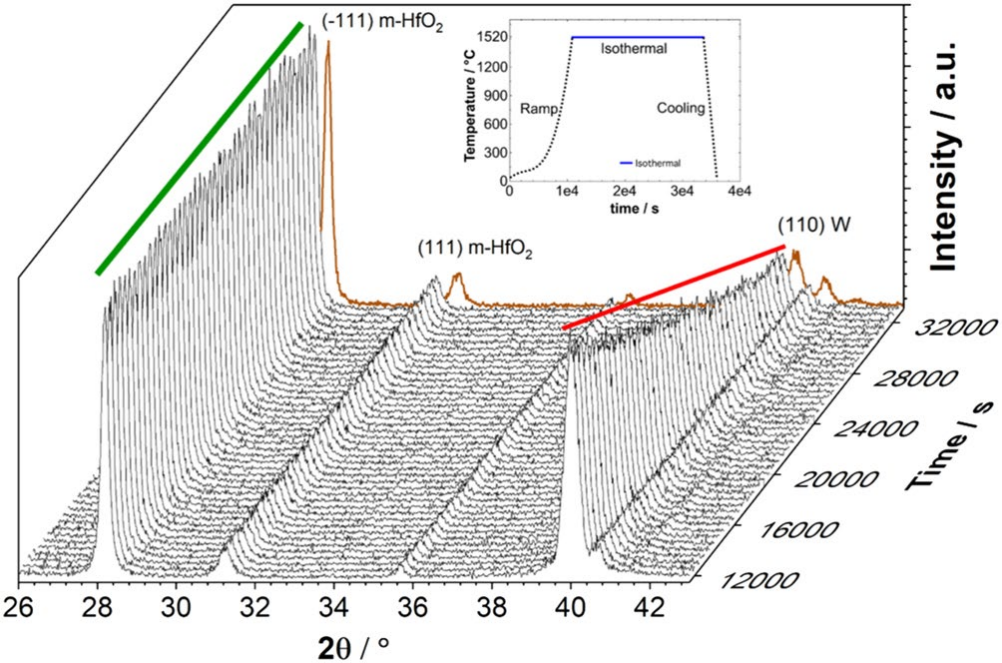
In-situ X-ray diffraction measurements of a 3-layer-system performed during isothermal annealing at 1520 °C for 6 h. The inset shows the whole annealing experiment with the isothermal part marked by a thick blue line. The (− 111) peak of the monoclinic HfO_2_ phase is unchanged, however, the (110) peak of the bcc W phase is decreased successively. The last diffractogram colored in brown is measured at room temperature, at the end of the annealing experiment.

The second and most important stage in the temperature excursion of the 3-layer-system is the isothermal process. Here, diffraction patterns are recorded as a function of time under constant temperature. These measurements yield significant information on the kinetics of degradation observed in the 3-layer-system. 2θ scans in the range of 26°–43° at 1520 °C spanning over 6 h are displayed in Fig. 4. There is a slow increase in 2θ values of both the (− 111) monoclinic HfO_2_ and (110) bcc W peaks. The peaks get sharper over time and the FWHM of both the peaks decrease due to grain coarsening. There is no indication of any phase or composition change appearing in the diffraction patterns, which is ideal for the application as high-temperature stable material. The most striking feature to observe is the intensity reduction of the (110) bcc W peak over time, while the (− 111) monoclinic HfO_2_ remains constant during the 6 h annealing period. After cooling down the 3-layer-system to room temperature, the diffractograms do not show any additional phase of HfO_2_ and it persists in the monoclinic phase. This is represented in the last scan (brown color, Fig. 4) of the sequence of diffraction patterns, in which a shift in 2θ value to higher angle is observed due to thermal contraction. It is important to mention that the final properties of the 3-layer system after in-situ and ex-situ experiments are similar, but it also indicates that the in-situ scans give us the tools to reveal information about internal and as well irreversible changes of structure and microstructure.

**Figure 5:**
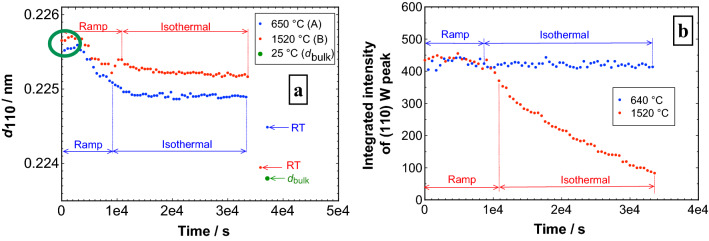
(**a**) Interplanar spacing *d*_110_ of bcc W in the 3-layer-system as a function of time during annealing up to 650 °C (blue markers) and 1520 °C (red markers). The duration of the isothermal part was about 6 h. The green ellipse highlight the initial increase in d-values due to thermal expansion below 200 °C. (**b**) Integrated intensity of (110) bcc W in 3-layer-systems annealed for 6 h at temperatures 650 °C (blue markers) and 1520 °C (red markers).

The time-dependent change of the interplanar spacing *d*_110_ of the bcc W phase allow us a first insight of the degradation process. Therefore, the influence at two annealing temperatures 650 °C (red markers) and 1520 °C (blue markers) was measured (Fig. 5a). Before annealing, the d-values measured at room temperature for the two samples are nearly the same, since the values are *d*_110_ = 0.2255 nm for the sample 650 °C and *d*_110_ = 0.2256 nm for sample 1520 °C. The small vertical offset (of less than 0.0001 nm) between the room temperature values is a systematic error caused by the initial height adjustments of the high-temperature stage before the start of the experiment. In general, the interplanar spacing *d*_110_ without any stress relaxation or other irreversible process increases with higher temperature during annealing. However, it is essential to compare steady- state d-values at two different fixed temperatures to understand the significance of grain growth, stress relaxation and recrystallization with respective to the high-temperature stability. Initially, there is a small increase of d-value until 200 °C the first five points (highlighted in green ellipse, Fig. 5a), this is due to the thermal expansion as the atomic mobility is still too small for stress relaxation. The observed thermal expansion of W film is 4 × 10^–6^ K^−1^ and it is in good agreement with bulk W^58^, i.e. 4.12 × 10^–6^ K^−1^. Using magnetron sputtering at low Ar pressure of 2 × 10^–3^ mbar, the films are most likely in a state of compressive stress^59^ as a result of atomic peening^60,61^. Thus, the growing film is densified by later arriving atoms having a mean kinetic energy in the order of 2–10 eV^62^.

Above 200 °C, until the onset of the isothermal state of the heating experiment, atomic mobility is now large enough to release stress, hence a drop in the d-value is observed. Later during the isothermal stage, there is initially a drop in d-values due to stress release, but later it is gradually counter-reacted by the thermal expansion and the d-values stabilize at 0.2248 nm for 650 °C sample and 0.2252 nm for 1520 °C sample. On cooling down the sample to room temperature at the end of the annealing experiment, the d-value of the sample annealed at 1520 °C almost reaches the tabled equilibrium value of bulk W *d*_110_ = 0.2238 nm (PDF card 00-004-0806). It is worthwhile to mention that the difference in d-values at room temperature after annealing at 1520 °C and 650 °C is increased, since the interplanar spacings are 0.2239 nm and 0.2244 nm, respectively. The sample annealed at 1520 °C shows the lower d-value and it is close to the indicated bulk equilibrium value, because almost all the stresses had been released upon annealing. Whereas the sample annealed at 650 °C lands at a higher d-value because there is still some stress present and not all of it had been released, thereby leading to an increased d-value compared to the bulk value. From the above observation it can be demonstrated that the nanocrystalline structure of bcc W is significantly altered after annealing at 1520 °C. For monoclinic HfO_2_, the interplanar spacing *d*_-111_ is also decreased during annealing but the changes are in general more moderate (Supplementary Fig. 3).

In order to assess the changes in the W layer during the in-situ annealing experiment, the integrated intensity (area under the diffraction peak) values of (110) bcc W peak annealed at 650 °C (blue markers) and 1520 °C (red markers) are shown in Fig. 5b. From quantitative analysis of powder mixtures, it is known that the integrated intensity of a diffracted peak for a particular phase depends on the concentration of the phase. This indicates that a relative change of integrated intensity of the (110) bcc W peak is proportional to a change of the volume fraction of the W present in the 3-layer-system. The integrated intensity of the sample annealed at 650 °C shows a constant behavior during the temperature ramp-up, and it remains relatively stable during the whole isothermal stage. Whereas in the sample annealed at 1520 °C, the integrated intensity remains relatively constant during the temperature ramp-up and then drastically decreases during the isothermal part of the annealing experiment. Any loss in the integrated intensity of (110) W peaks directly indicates a loss in volume fraction of W in the 3-layer-system. A change in roughness could also explain a reduction in peak intensity, but under such circumstances, the intensities of all occurred phases for HfO_2_ and W should be reduced. Clearly, this is not observed for the intensity of the monoclinic HfO_2_ phase (Fig. 4), that appears fairly constant over the whole isothermal stage. The calculated volume fraction of the W disappearing in the 20 nm thick W layer sandwiched between two HfO_2_ layers is estimated to be 79% at the end of the 6 h isothermal stage at 1520 °C. Further experimental results at various annealing temperatures are exploited to determine an activation energy for this structural degradation, which is described later.

Microstructural information like grain size is as well contained in the broadening of X-ray diffraction peaks. The grain size changes of monoclinic HfO_2_ during heating ramp and isothermal stages at 650 °C and 1520 °C are shown in Supplementary Fig. 4. The grain size value is calculated from the (− 111) monoclinic HfO_2_ peak using the Scherrer formula^63^, which gives us an estimate of the average grain size in the out-of-plane direction. A decrease in FWHM of a diffraction peak on annealing compared to the as prepared sample indicates grain growth in the layer. An increase in grain growth is observed by a factor of two for the 3-layer-system annealed at 1520 °C compared to the as prepared sample. However no significant increase in grain growth is observed for the 3-layered-system annealed at 650 °C. The growth process of the W grains is in principle similar (Supplementary Fig. 5), however in the isothermal part at 1520 °C, the atomic loss in the W grains is predominant due to sublimation of W oxide, which is discussed in detail later.

**Figure 6:**
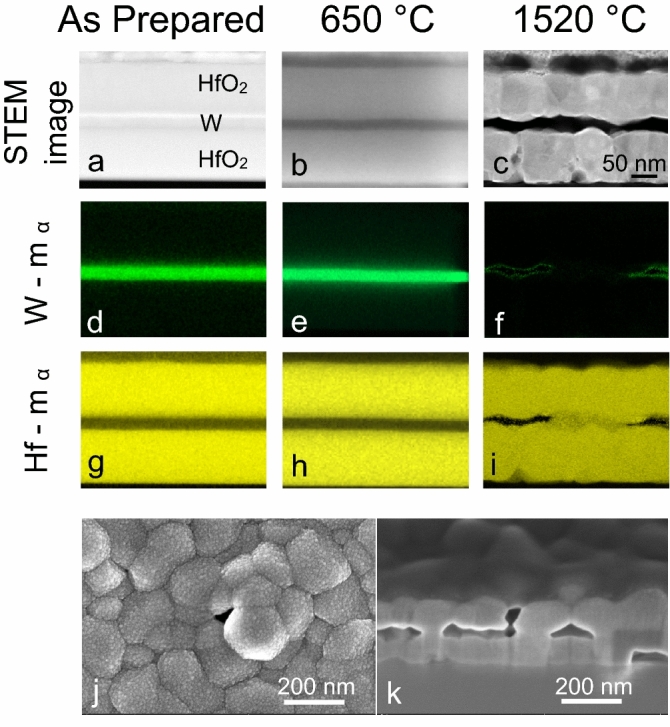
STEM-EDS analysis of the 3-layer-system, as-prepared, annealed at 650 °C and 1520 °C for 6 h. (**a**–**c**) STEM-HAADF images, (**d**–**f**) W-Mα elemental mapping, (**g**–**i**) Hf-Mα elemental mapping. These images have the same scale as indicated in image (**c**). SEM images of the 3-layer-system annealed at 1520 °C for 40 h: (**j**) morphology of the top HfO_2_ layer with a void in the middle and (**k**) cross section view along the interfaces across the same void (FIB section).

To achieve a deeper understanding of the structural changes in W and HfO_2_ layers due to the in-situ annealing experiments, TEM-EDS characterizations on the as prepared and annealed samples were performed. In the case of the as prepared sample, the STEM-high-angle annular dark-field (STEM-HAADF) image and the elemental mapping (Fig. 6a,d,g) show a distinct 3-layer-system. After annealing at 650 °C for 6 h, there are no modifications in the layer stack (Fig. 6b,e,h), the interface between W and HfO_2_ layers appears to be unchanged. No grain boundaries were seen in the studied TEM lamella. Figure 6a,b were captured at different camera lengths, this results in different diffraction contrast, leading to bright and dark lines above and below the W layer. No additional phase of W is observed in the EDS mapping. On the contrary, after 1520 °C and 6 h, significant changes in morphology occur (Fig. 6c). An increase in the interface roughness of the HfO_2_ layers is clearly visible and the interface becomes wavy in contrast to the sharp interfaces prior to annealing. Individual grains are clearly seen with distinct grain boundaries due to the increase in grain size. The EDS mapping of W (Fig. 6f) shows a very weak presence of W in the layer, which corroborates the XRD results indicating the reduction of W volume fraction (Fig. 5b) after annealing at 1520 °C. In addition, the EDS mapping of Hf (Fig. 6i) after annealing at 1520 °C for 6 h confirms the presence of Hf in the HfO_2_ layer and no diffusion of Hf into the W layer.

In order to understand the root cause for the disappearance of the W from the sandwiched layer, further heating experiments were conducted to estimate the total time required for the entire W layer to disappear from the 3-layer-system and to investigate the remaining morphology. A 3-layer-system was annealed for 40 h at 1520 °C for this purpose. The top surface and cross-section SEM images of the 3-layer-system annealed at 1520 °C for 40 h are shown in (Fig. 6j,k). The presence of voids distributed on the top HfO_2_ layer is visible, which is in good agreement to early studies on crystallization of HfO_2_ single films^56^. In our system, they extend through the entire thickness of the first HfO_2_ layer, illustrated by the cross section view through the same void shown in Fig. 6k. It is also noteworthy to observe the complete absence of W in the 3-layer-system sample. The voids present in the HfO_2_ layer act as a transport channel for the residual O_2_ molecules in the heating chamber to reach the W layer, resulting in oxidation and the formation of volatile WO_x_^64^. As seen previously for the 6 h annealed 3-layer-system, a similar trend in the loss of intensity from the (110) W peak occurred, and after 20 h (see below in Fig. 8a), the intensity of the W peak was reduced considerably and was almost in-line with the background in the measured diffraction pattern. The volatile WO_x_ sublimate rapidly at 1520 °C, leaving the layer through the same transport channels. Consequently, no WO_x_ peaks are visible in the diffractograms. A plain sapphire “witness” substrate was therefore placed on the wall of the heating chamber close to the outlet of the vacuum pump to collect any sublimated material via re-deposition. The redeposited layer on the witness sample can be identified as WO_2_ from the diffraction pattern (Supplementary Fig. 6). The possibility of W obtaining O_2_ from HfO_2_ can be excluded, as this reduction reaction would either result in the formation of pure Hf or result in sub-stoichiometric HfO_2_. No Hf peaks are observed in any of our in-situ X-ray diffraction experiments. In addition, and complementarily, characterization by Rutherford backscattering spectrometry (RBS) (Supplementary Fig. 7) confirms no presence of pure Hf but the appearance of slightly over stoichiometric HfO_2+x_ and especially the loss of W.

**Figure 7:**
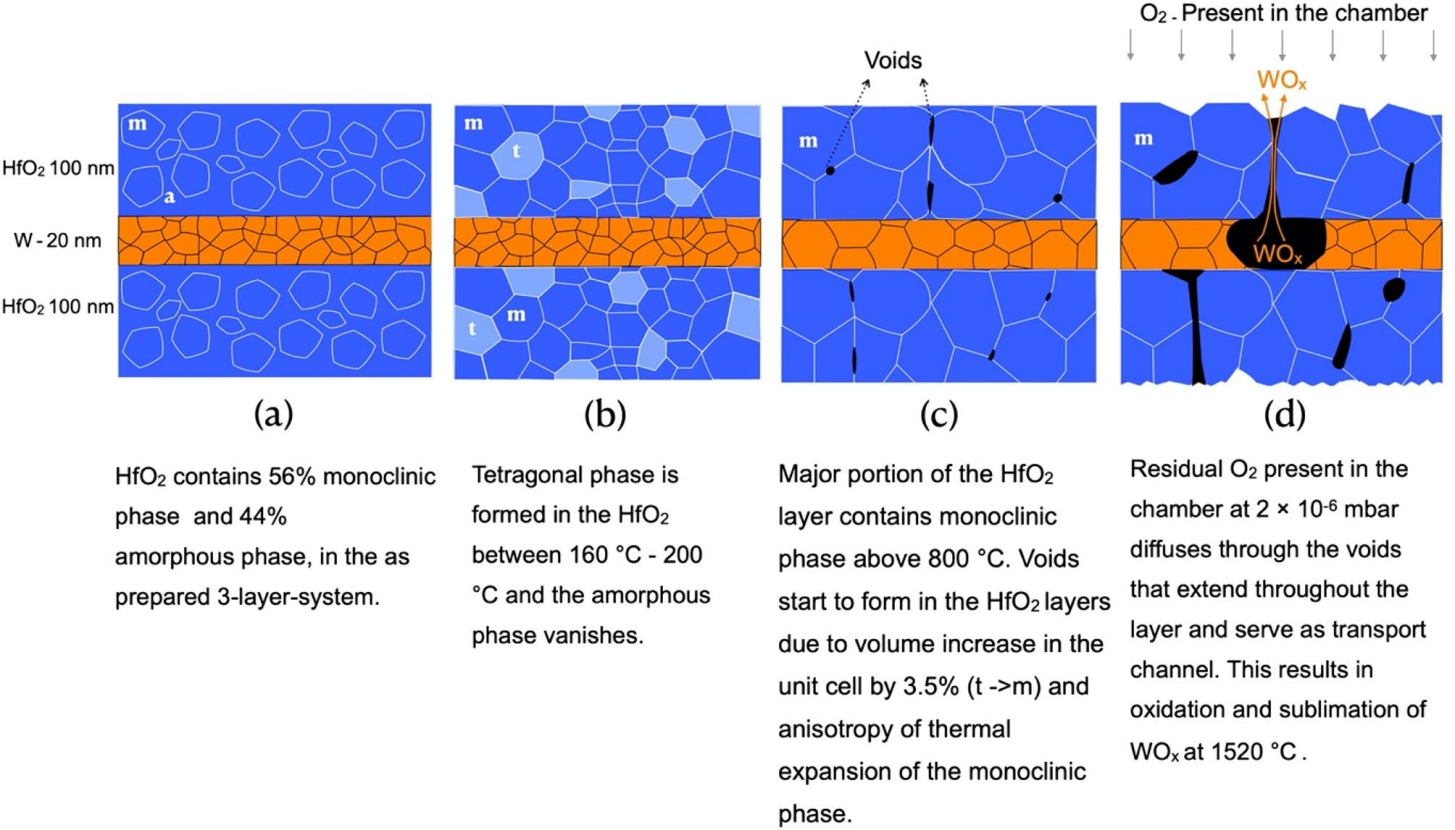
Mechanisms of channel formation in the HfO_2_ layers and loss of W in the 3-layer-system.

The complex oxidation mechanism of W is extensively discussed in literature^65–74^. It is possible to split the oxidation mechanism of W into four different zones up to a temperature of 1500 °C depending on the partial pressure of O_2_. The first zone is comprised of heating experiments conducted until 500 °C in the partial pressure of O_2_ higher than 100 mbar^65^, an increase in the mass of the bulk W samples is observed during heating experiments. The increase in mass is attributed to the formation of an oxide layer on top of the sample. The oxidation of W is an exothermic process, where heat is liberated when W and O_2_ react to form WO_x_ having a negative enthalpy of formation^75^ and the oxidation process is diffusion-controlled, requiring an activation energy of 1.92 eV. The second zone comprises the temperature range between 500 °C until 1100 °C in an O_2_ partial pressure of 100 mbar^66^, where complete oxidation of the W sample occurs. Initially, the oxidation process follows a parabolic rate law due to the diffusion of O_2_ through the already formed WO_3_ top layer. Later, the formed oxide cracks due to stress exposing the underlying metal and then the oxidation process follows a linear rate law. The activation energy for oxidation in this temperature range is 1.4 eV. The third zone of oxidation can be distinguished by increasing the temperature to 1250 °C at 50 mbar O_2_ pressure, where all the O_2_ reacts to form volatile WO_3_ and no formation of any oxide layers were visible on the sample surface. Thus, the rate of WO_3_ sublimation was quicker than the rate for the formation of a solid oxide layer above 1250 °C. In our previous annealing experiment^27^ performed at 1200 °C and in a vacuum of 2 × 10^–2^ mbar, the W present in the multilayered metamaterial was oxidized and the presence of an oxide layer of WO_2_ on top of the first W layer was confirmed by XRD and HRTEM characterization. The fourth zone of oxidation mechanism can be distinguished for temperatures between 1250 and 2300 °C in a vacuum less than or equal to 1 × 10^–5^ mbar. Only weight loss is observed in all the W samples without the formation of a solid oxide layer, as the concentration of O_2_ atoms is relatively small. The activation energy^76^ in this zone can vary from 0.6 to 0.86 eV depending on the temperature.

In our current in-situ annealing experiments conducted at 2 × 10^–6^ mbar, the diffractograms do not show any peaks of WO_x_ during the entire heat treatment cycle (Figs. 3 and 4). Based on the results of the 40 h annealing experiment, further annealing experiments, for a duration of 20 h at different temperatures between 650 and 1520 °C, were performed. The objective of the latter experiments was to determine the temperature range where the voids start to originate in the HfO_2_ layers. It is observed from the SEM images (Supplementary Fig. 8) that voids appear on all samples annealed above 800 °C. This temperature range can be related to the transformation of the tetragonal phase to the stable monoclinic phase discussed previously. A change in structure from tetragonal to monoclinic results in a volume increase accompanied by shear strain^30^. It is also to be noted that the symmetry of the structure is reduced from tetragonal (a = b ≠ c, ß = 90°) to monoclinic (a ≠ b ≠ c, ß ≠ 90°). This results in anisotropic behavior of thermal expansion, where the minimum and maximum thermal expansion values do not lie in the lattice directions for a monoclinic structure. The coefficient of thermal expansion values of the monoclinic phase range between 8 × 10^–6^ K^−1^ to 32 × 10^–6^ K^−1^ at 1750 °C^77^. Therefore, the tetragonal-to-monoclinic phase transformation in HfO_2_ and the anisotropic nature of the monoclinic phase results in the formation of voids between the HfO_2_ grains in the top HfO_2_ layer. The mechanism of void formation and the oxidation of the W layer are summarized in Fig. 7.

**Figure 8.**
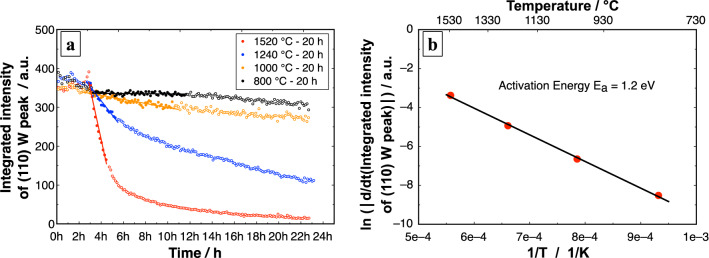
(**a**) Integrated intensity of (110) W peak annealed at different temperatures for 20 h are shown. The slopes marked by the filled circles for each temperature are displayed as time derivatives in the logarithmic plot (**b**) in order to calculate the activation energy from its slope using a linear fit. (**b**) Arrhenius plot for the oxidation of W in the 3-layer-system.

The integrated intensity of the (110) bcc W peak of the sample annealed at 800 °C decrease only slightly after 20 h (Fig. 8a). On increasing the temperature, the loss of intensity is observed for the sample annealed at 1000 °C and 1240 °C. Moreover, the sample annealed at 1520 °C shows a sharp decrease in intensity during the initial 3 h of the isothermal stage and later the process slows down to a reduced rate. The change in the volume fraction is estimated by calculating the slopes for the drop in integrated intensity of the (110) bcc W peak (filled circles in the plots were used for the calculation of slope, Fig. 8a). This thermally-activated time-dependent process is represented by an Arrhenius plot (Fig. 8b). The change in the integrated intensity of the (110) bcc W peak over time is expressed as a function of the inverse of the annealing temperature. An activation energy of 1.2 eV is calculated for O_2_ to penetrate through the transport channels, oxidize the W and sublimate as volatile WO_x_. Becker et al.^70^ calculated an activation energy of 1.1 eV, which is in good agreement with our experiments. They performed an estimation for the conversion of WO_3_ at a pressure of 1 × 10^–6^ mbar and in the temperature range between 920 to 1320 °C, when O_2_ molecules strike the surface of a W ribbon. In our experiments, the rate of W loss can also depend on the number and the size of pores in HfO_2_. The HfO_2_ heated to higher temperatures can provide more or larger channels for O_2_ attack and thus increased rate of W loss. Thus, the obtained activation energy can be alternatively linked to the surface diffusion of HfO_2_ that leads to grain growth and void formation.

At a temperature below the phase transformation in HfO_2_, no sign of degradation is observed in the W layer. The tetragonal phase was retained on cooling the 3-layer-system annealed at 300 °C for 20 h, back to room temperature. It is worthwhile to mention that an annealing experiment at 700 °C and over a time of 100 h do not cause any remarkable degradation to the sandwiched W layer, only above this temperature, which coincides with the tetragonal to monoclinic phase transformation in HfO_2_, we observe the degradation of W in our 3-layer-system.

## Conclusion

The thermal stability and durability of the selective emitters at high-temperature are the essential prerequisites for the effective functioning of the TPV system. In order to meet these requirements, a 3-layer-system comprising of HfO_2_/W/HfO_2_ was fabricated using magnetron sputtering. The high-temperature stability of the 3-layer-system was evaluated by performing in-situ XRD annealing experiments at various annealing temperatures between 300 and 1520 °C at three different periods 6 h, 20 h, and 40 h and at vacuum pressures of 3 × 10^–6^ mbar.

The disappearance of W in the 3-layer-system is validated by the drop in the integrated intensity of the (110) bcc W peak during the isothermal part of all the annealing experiment performed over 1000 °C. The mechanism behind the degradation of the W layer begins with the formation of voids in the HfO_2_ layers that acts later as transport channels. The tetragonal-to-monoclinic phase transformation results in volume increase, which is accompanied by the anisotropic nature of the monoclinic HfO_2_ phase, and culminates in the formation of voids. Residual O_2_ present in the heating chamber reaches the W layer through the transport channels and forms volatile oxides that sublimate rapidly. An activation energy of 1.2 eV is determined for the oxidation of W in the 3-layer-system through the transport channels, by performing additional annealing experiments between 800 and 1520 °C for 20 h.

Although the combination of W/HfO_2_ excel exceptionally to meet the optical properties of the selective emitter, HfO_2_ fails to act as a good protective layer above 1000 °C. The thermal stability and durability at high-temperature of the 3-layer-systems as well as multilayered metamaterials could be further enhanced by reducing grain growth and avoiding a phase transformation like the tetragonal-to-monoclinic transition observed in HfO_2_, thereby preventing the formation of voids in the oxidic dielectric layers. We will investigate various combinations of metals for instance suitable W alloys and dielectrics such as Y-stabilized HfO_2_ in order to achieve a higher working temperature and longer lifetime of selective emitters for TPV systems.

## Methods

### Thin-film deposition using magnetron sputtering

All 3-layer-systems used for annealing experiments were prepared by magnetron sputtering, using our in-house designed sputtering facility at the Helmholtz-Zentrum Geesthacht. The chamber was evacuated to a base pressure below 1 × 10^–7^ mbar before the start of the thin film deposition process. The W and HfO_2_ sputtering targets (Sindlhauser Materials GmbH) had a purity of 99.95% and were 3″ in diameter. The 3-layer-system HfO_2_/W/HfO_2_ was deposited on a sapphire substrate 10 mm × 10 mm × 0.5 mm (purchased from CrysTec GmbH) having a (1–102) orientation and thermal expansion coefficients of 5.3 × 10^–6^ K^−1^ (parallel to c-axis) and 4.5 × 10^–6^ K^−1^ (perpendicular to c-axis). The substrates are mounted on a custom-made holder equipped with circulating cooling water system and are rotated at a rate of 1 Hz. The temperature of the cooling water is maintained below 20 °C throughout the deposition of the 3-layer-system. The distance between the target and the substrate is 14 cm. The calibration of the individual deposition rate of W and HfO_2_ was done separately by deposition of single layers on silicon substrates 20 mm × 60 mm × 0.7 mm having a (100) orientation parallel to the substrate normal. Argon gas of purity 99.99999% at a flow rate of 28 sccm was used as sputtering gas and the sputtering pressure was maintained at 2 × 10^–3^ mbar. The thin-film preparation of the W films was done using a DC power of 120 W, with a deposition rate of 0.07 nm s^−1^, whereas HfO_2_ films were deposited using RF power of 400 W, which yielded in a deposition rate of 0.15 nm s^−1^.

### In-situ X-ray diffraction experiments

A diffractometer, equipped with a specific high-temperature chamber for annealing up to 2000 °C (Anton Paar—HTK 2000N), and a state-of-the-art position sensitive detector (Lynxeye XE-T, Bruker AXS) with an energy resolution of less than 380 eV was used to perform all the annealing experiments. Thermal expansion calibration experiments were performed with sapphire substrates up to 1900 °C, in temperature steps of 100 °C, and using a heating rate of 25 °C min^−1^. A full pattern fit was performed for a 2θ range from 50° to 125° and the lattice parameters of the sapphire substrate at every 100 °C interval were refined using the Rietveld method (TOPAS)^78^. The temperatures were later validated from the thermal expansion coefficients determined by Touloukian et al.^58^. The heating chamber was evacuated to a pressure less than 3 × 10^–6^ mbar before every annealing experiment. The z-height of the heating stage was adjusted better than 10 µm by using the sapphire substrate peak positions according to Powder Diffraction File (PDF)^79^ card 01-082-1468. The XRD 2θ scans were measured from 20° to 120°, with an increment of 0.02° and a time of 0.1 s per step, using Cu Kα1 radiation (*λ* = 0.15406 nm). The area of the sample that is exposed to X-rays is 3 mm × 10 mm. The annealing experiments were performed with three different dwell times of 6 h, 20 h and 40 h. The heating rate during the ramp was 120 °C min^−1^, and the cooling was done at a rate of 60 °C min^−1^. The diffraction patterns were recorded at different temperatures during the ramp part and at a time interval of 8 min during the isothermal part of the experiments. During analysis of the diffraction patterns, position, width and integrated intensity (net area) of the diffraction peaks were determined in order to evaluate interplanar spacing, grain size and volume fraction of a distinct phase, respectively. Grain size *t* in the out-of-plane direction of the W and HfO_2_ layers is calculated using the Scherrer equation^63^:

1 $$t = K \lambda { }/(B cos\theta_{{\text{\rm B}}} ),$$

where, *t* is the grain size, *K* is a dimensionless shape factor (*K* = 0.9)^63^, *λ* is the wavelength of X-rays (for Cu-radiation: *λ* = 0.15406 nm), *B* is the full width at half maxima (FWHM) of the peak, *θ*_B_ is the glancing angle (radians), which is the half of the Bragg angle. Instead of grain size, the term crystallite size^80^^,^^81^ is widely used in power diffraction and sintering of ceramics^82^. Furthermore, the interplanar spacing *d*_hkl_ is determined from the measured peak position 2*θ*_hkl_ in the diffraction patterns using Bragg’s condition, which results in the following formula:

2 $$d_{{{\text{hkl}}}} = \lambda /(2 sin\theta_{{{\text{hkl}}}} ).$$

The layer thickness of W and HfO_2_ were determined precisely by means of X-ray reflectometry (XRR), using Cu K_α1_ radiation (*λ* = 0.15406 nm). Layer properties were evaluated by fitting simulations to experimental reflectivity scans with the Leptos R software package (Bruker AXS).

### TEM and EDS characterization

The cross-sectional transmission electron microscopy (TEM) samples, and cross-sections for scanning electron microscopy (SEM) imaging were prepared with a FEI Helios G3 UC focused ion beam scanning electron microscope (FIB-SEM). Both the cross-sectional TEM lamellae and cross-sections were prepared using 30 keV Ga^+^ ion beam and different beam currents (9 nA to 40 pA). Secondary electron (SE) images of the cross-sections were taken under low keV (2 keV) electron beam and high-resolution immersion mode using a through lens detector (TLD). To prevent charging during the FIB-SEM study, the sample was coated with a thin layer of carbon by means of a carbon thread evaporator. A FEI Talos F200X TEM equipped with a four-quadrant silicon drift detector system for energy dispersive X-Ray spectroscopy [Super-X(FEI)] system was used for taking high angle annular dark field (HAADF) images and to perform EDS analysis on 10 µm × 100 nm lamellae. Spectrum images (SI) were acquired using a 1 nA beam current and dwell time of 10 µs per pixel. Resolution was 512 × 512 pixels with a pixel size of 766 pm. To get a better signal-to-noise ratio integrated intensities of over 1000 drift corrected SI were used.

## Supplementary Information

Supplementary Information.

## References

[1] Brockway PE, Owen A, Brand-Correa LI, Hardt L (2019). Estimation of global final-stage energy-return-on-investment for fossil fuels with comparison to renewable energy sources. Nat. Energy.

[2] Swanson RM (1979). A proposed thermophotovoltaic solar energy conversion system. Proc. IEEE.

[3] Bauer T (2011). Thermophotovolatics..

[4] Omair Z (2019). Ultraefficient thermophotovoltaic power conversion by band-edge spectral filtering. Proc. Natl. Acad. Sci. U. S. A..

[5] Ganapati, V., Xiao, T. P. & Yablonovitch, E. Ultra-efficient thermophotovoltaics exploiting spectral filtering by the photovoltaic band-edge. 1–14 (2016).

[6] Boriskina SV (2016). Roadmap on optical energy conversion. J. Opt..

[7] Swanson, R. M. Silicon photovoltaic cells in thermophotovoltaic energy conversion. 70–77 (1978).

[8] Chirumamilla M (2017). Large-area ultrabroadband absorber for solar thermophotovoltaics based on 3D titanium nitride nanopillars. Adv. Opt. Mater..

[9] Crowley CJ, Elkouh NA, Murray S, Chubb DL (2005). Thermophotovoltaic converter performance for radioisotope power systems. AIP Conf. Proc..

[10] Datas A, Martí A (2017). Thermophotovoltaic energy in space applications: Review and future potential. Sol. Energy Mater. Sol. Cells.

[11] Wang H (2020). Thermal emission-enhanced and optically modulated radioisotope thermophotovoltaic generators. Energy Technol..

[12] Nelson RE (2003). A brief history of thermophotovoltaic development. Semicond. Sci. Technol..

[13] Coutts TJ (1999). Review of progress in thermophotovoltaic generation of electricity. Renew. Sustain. Energy Rev..

[14] Ferrari C, Melino F, Pinelli M, Spina PR, Venturini M (2014). Overview and status of thermophotovoltaic systems. Energy Proc..

[15] Wang Y, Liu H, Zhu J (2019). Solar thermophotovoltaics: Progress, challenges, and opportunities. APL Mater..

[16] Molesky S, Dewalt CJ, Jacob Z (2013). High temperature epsilon-near-zero and epsilon-near-pole metamaterial emitters for thermophotovoltaics. Opt. Express.

[17] Li J, Hossain MDM, Jia B, Buso D, Gu M (2010). Three-dimensional hybrid photonic crystals merged with localized plasmon resonances. Opt. Express.

[18] Rephaeli E, Fan S (2009). Absorber and emitter for solar thermo-photovoltaic systems to achieve efficiency exceeding the Shockley–Queisser limit. Opt. Express.

[19] Chan DLC, Soljačić M, Joannopoulos JD (2006). Thermal emission and design in 2D-periodic metallic photonic crystal slabs. Opt. Express.

[20] Liu X (2011). Taming the blackbody with infrared metamaterials as selective thermal emitters. Phys. Rev. Lett..

[21] Sakakibara R (2019). Practical emitters for thermophotovoltaics: A review. J. Photonics Energy.

[22] Pfiester NA, Vandervelde TE (2017). Selective emitters for thermophotovoltaic applications. Phys. Status Solidi Appl. Mater. Sci..

[23] Woolf DN (2018). High-efficiency thermophotovoltaic energy conversion enabled by a metamaterial selective emitter. Optica.

[24] Planck, M. *The Theory of Heat Radiation*. (P. Bankiston’s Son & Co., 1914).

[25] Chubb, D. Fundamentals of thermophotovolatic energy conversion. *Elsevier Sci.* **530** (2007).

[26] Bett AW, Sulima OV (2003). GaSb photovoltaic cells for applications in TPV generators. Semicond. Sci. Technol..

[27] Chirumamilla M (2019). Metamaterial emitter for thermophotovoltaics stable up to 1400 °C. Sci. Rep..

[28] Shabalin, I. L. *Ultra-high temperature materials I: Carbon (graphene/graphite)*. *Ultra-High Temperature Materials I: Carbon (Graphene/Graphite) and Refractory Metals* (2014). 10.1007/978-94-007-7587-9.

[29] Hlavac J (1982). Melting temperatures of refractory oxides: Part I. Pure Appl. Chem..

[30] Wang J, Li HP, Stevens R (1992). Hafnia and hafnia-toughened ceramics. J. Mater. Sci..

[31] Rinnerbauer V (2013). High-temperature stability and selective thermal emission of polycrystalline tantalum photonic crystals. Opt. Express.

[32] Silva-Oelker G, Jerez-Hanckes C, Fay P (2018). Study of W/HfO_2_ grating selective thermal emitters for thermophotovoltaic applications. Opt. Express.

[33] DyachenkoDyachenko PN (2016). Controlling thermal emission with refractory epsilon-near-zero metamaterials via topological transitions. Nat. Commun..

[34] Arpin KA (2013). Three-dimensional self-assembled photonic crystals with high temperature stability for thermal emission modification. Nat. Commun..

[35] Yokoyama T (2016). Spectrally selective mid-infrared thermal emission from molybdenum plasmonic metamaterial operated up to 1000 °C. Adv. Opt. Mater..

[36] Chang, C. C. *et al.* High-temperature refractory metasurfaces for solar thermophotovoitaic energy harvesting. In *2019 Conf. Lasers Electro-Optics, CLEO 2019—Proc.* (2019). 10.23919/CLEO.2019.8750285.

[37] Kim JH, Jung SM, Shin MW (2019). Thermal degradation of refractory layered metamaterial for thermophotovoltaic emitter under high vacuum condition. Opt. Express.

[38] Kohiyama A, Shimizu M, Yugami H (2016). Unidirectional radiative heat transfer with a spectrally selective planar absorber/emitter for high-efficiency solar thermophotovoltaic systems. Appl. Phys. Express.

[39] Shimizu M, Kohiyama A, Yugami H (2015). High-efficiency solar-thermophotovoltaic system equipped with a monolithic planar selective absorber/emitter. J. Photonics Energy.

[40] Lee H-J (2013). Hafnia-plugged microcavities for thermal stability of selective emitters. Appl. Phys. Lett..

[41] Han S, Shin J-H, Jung P-H, Lee H, Lee BJ (2016). Broadband solar thermal absorber based on optical metamaterials for high-temperature applications. Adv. Opt. Mater..

[42] Chirumamilla M (2016). Multilayer tungsten-alumina-based broadband light absorbers for high-temperature applications. Opt. Mater. Express.

[43] Dyachenko PN (2015). Tungsten band edge absorber/emitter based on a monolayer of ceramic microspheres. Opt. Express.

[44] Chou JB (2014). Enabling ideal selective solar absorption with 2D metallic dielectric photonic crystals. Adv. Mater..

[45] Stelmakh, V., Chan, W. R., Joannopoulos, J. D., Soljacic, M. & Celanovic, I. Sputtered tantalum photonic crystal coatings for high-temperature energy conversion applications. *IEEE-NANO 2015 15th Int. Conf. Nanotechnol.* **2**, 1134–1137 (2015).

[46] Stelmakh, V. *et al.* Evolution of sputtered tungsten coatings at high temperature. *J. Vac. Sci. Technol. A Vacuum Surf. Film.* **31**, 061505 (2013).

[47] Hauffe. High temperature oxidation of metals. P. Kofstad John Wiley & Son, New York 1966, 340 S. *Mater. Corros.* **18**, 956–957 (1967).

[48] Ohring, M. Chapter 3—thin-film evaporation processes. In *Materials Science of Thin Films (Second Edition)* (ed. Ohring, M.) 95–144 (Academic Press, Cambridge, 2002). 10.1016/B978-012524975-1/50006-9.

[49] Lassner, E. & Schubert, W.-D. *Tungsten: Properties, Chemistry, Technology of the Element, Alloys, and Chemical Compounds Erik Lassner and Wolf-Dieter Schubert*. *Tungsten. Properties, Chemistry, Technology of the Element, Alloys, and Chemical Compounds* (1999). 10.1007/978-1-4615-4907-9.

[50] Huminik, J. *High-Temperature Inorganic Coatings*. (Reinhold Publishing Corporation, 1963).

[51] Ohring, M. Chapter 5—plasma and ion beam processing of thin films. In *Materials Science of Thin Films (Second Edition)* (ed. Ohring, M.) 203–275 (Academic Press, Cambridge, 2002). 10.1016/B978-012524975-1/50008-2.

[52] Thornton JA (1974). Influence of apparatus geometry and deposition conditions on the structure and topography of thick sputtered coatings. J. Vac. Sci. Technol..

[53] Thornton JA (1977). High rate thick film growth. Ann. Rev. Mater. Sci..

[54] Salamon K (2013). Structure and morphology of magnetron sputtered W films studied by X-ray methods. J. Phys. D. Appl. Phys..

[55] Thompson CV (1990). Grain growth in thin films. Annu. Rev. Mater. Sci..

[56] Ushakov SV (2004). Crystallization in hafnia- and zirconia-based systems. Phys. Status Solidi Basic Res..

[57] Zhao, C. *et al.* Thermal stability of high k layers. In *Novel materials and processes for advanced CMOS* (eds. Gardner, M. I. *et al.*) **745**, 9–14 (Materials Research Society, 2003).

[58] Touloukian, Y. S., Kirby, R. K., Taylor, R. E. *Thermo-Physical Properties of Matter*. (Plenum Press, 1977).

[59] Hu SM (1991). Stress-related problems in silicon technology. J. Appl. Phys..

[60] D’Heurle FM, Harper JME (1989). Note on the origin of intrinsic stresses in films deposited via evaporation and sputtering. Thin Solid Films.

[61] Davis CA (1993). A simple model for the formation of compressive stress in thin films by ion bombardment. Thin Solid Films.

[62] Ohring, M. Chapter 4—Discharges, plasmas, and ion–surface interactions. In *Materials Science of Thin Films (Second Edition)* (ed. Ohring, M.) 145–202 (Academic Press, Cambridge, 2002). 10.1016/B978-012524975-1/50007-0.

[63] Cullity, B. *Elements of X-Ray Diffraction*. (Addison-Wesley Publishing Company Inc., 1978).

[64] Bergeron CG, Tennery VJ, Friedberg AL (1961). Reaction studies of ceramic-coated tungsten. J. Am. Ceram. Soc..

[65] Gulbransen EA, Wysong WS (1947). Thin oxide films on tungsten. J. Phys. Chem..

[66] Gulbransen EA, Andrew KF (1960). Kinetics of the oxidation of pure tungsten from 500° to 1300°C. J. Electrochem. Soc..

[67] Gulbransen EA, Andrew KF, Brassart FA (1964). Kinetics of oxidation of pure tungsten, 1150°–1615°C. J. Electrochem. Soc..

[68] Blackburn PE, Hoch M, Johnston HL (1958). The vaporization of molybdenum and tungsten oxides. J. Phys. Chem..

[69] Anderson, H. U., University of California, B. & Laboratory, L. R. Kinetic studies of the reactions occurring between tungsten and gases at low pressures and high temperatures. (1962).

[70] Becker JA, Becker EJ, Brandes RG (1961). Reactions of oxygen with pure tungsten and tungsten containing carbon. J. Appl. Phys..

[71] Tompkins, H. G. Adsorption on metal surfaces. *A User’s Guide to Ellipsometry* 196–212 (1993). 10.1016/b978-0-12-693950-7.50022-6.

[72] Singleton JH (1966). Interaction of oxygen with hot tungsten. J. Chem. Phys..

[73] Schissel PO, Trulson OC (1965). Mass-spectrometric study of the oxidation of tungsten. J. Chem. Phys..

[74] Ackermann RJ, Rauh EG, Thorn RJ, Cannon MC (1963). A thermodynamic study of the thorium-oxygen system at high temperatures. J. Phys. Chem..

[75] Barin I (1995). Thermochemical data of pure substances. Thermochem. Data Pure Substances.

[76] Urie H (1956). University of California Radiation Laboratory. Ind. Eng. Chem..

[77] Haggerty RP, Sarin P, Apostolov ZD, Driemeyer PE, Kriven WM (2014). Thermal expansion of HfO_2_ and ZrO_2_. J. Am. Ceram. Soc..

[78] Coelho AA (2018). TOPAS and TOPAS-Academic: An optimization program integrating computer algebra and crystallographic objects written in C++. J. Appl. Crystallogr..

[79] Gates-Rector S, Blanton T (2019). The powder diffraction file: A quality materials characterization database. Powder Diffr..

[80] Klug, H. P. & Alexander, L. E. *X-ray diffraction procedures for polycrystalline and amorphous materials/Harold P. Klug, Leroy E. Alexander*. (Wiley, New York, 1974).

[81] Cullity, B. D. & Stock, S. R. *Elements of X-ray Diffraction, Third Edition*. (Prentice-Hall, Upper Saddle River, 2001).

[82] Kingery, W. D., Uhlmann, D. R. & Bowen, H. K. *Introduction to ceramics/W. D. Kingery, H. K. Bowen, D. R. Uhlmann*. (Wiley, New York, 1976).

