# Supplementary material for: Structural degradation of tungsten sandwiched in hafnia layers determined by in-situ XRD up to 1520 °C
Source: Sci Rep. 2021 Feb 8;11:3330. doi: 10.1038/s41598-021-82821-0 (PMC7870937; doi:10.1038/s41598-021-82821-0)
Supplement: Supplementary file 1 — Supplementary Information. [file 41598_2021_82821_MOESM1_ESM.docx]

**Supplementary Information**

Structural degradation of tungsten sandwiched in hafnia layers determined by in-situ XRD up to 1520 °C

Gnanavel Vaidhyanathan Krishnamurthy^1*^, Manohar Chirumamilla^2^, Surya Snata Rout^3^, Kaline P. Furlan^4^, Tobias Krekeler^3^, Martin Ritter^3^, Hans-Werner Becker^5^, Alexander Yu Petrov^2^, Manfred Eich^1,2^, and Michael Störmer^1^.

^1^ Institute of Materials Research, Helmholtz-Zentrum Geesthacht, Max-Planck-Strasse 1, 21502 Geesthacht, Germany.

^2^ Institute of Optical and Electronic Materials, Hamburg University of Technology, Eissendorfer Strasse 38, Hamburg 21073, Germany.

^3^ Electron Microscopy Unit, Hamburg University of Technology, Eissendorfer Strasse 42, Hamburg 21073, Germany.

^4^ Institute of Advanced Ceramics, Hamburg University of Technology (TUHH), Denickestraße 15, 21073 Hamburg, Germany.

^5^ Central Unit for Ionbeams and Radionuclides, Ruhr-Universität Bochum, Universitätstraße 150, 44801 Bochum.

**Supplementary Figure 1**. XRD diffractogram of the 3-layer-system at 300 °C. The diffraction pattern of the confirmed tetragonal phase is shown.

The figure above shows the diffractogram of the 3-layer-system at 300 °C containing information from the tetragonal phase (PDF card 01-070-2831) and cubic phase (PDF card 01-078-5756) of HfO_2_. The peak positions for both the tetragonal and cubic phases are very close to each other. The tetragonal phase of HfO_2_ can be distinctly confirmed by observing the (004) peak at 72.6°. The diffraction pattern is consistent for all the 3-layer-systems reported in the article.

**Supplementary Figure 2**. In-situ X-ray diffraction measurements of a 3-layer-system performed during isothermal annealing at 300 °C for 20 h. The inset shows the whole annealing experiment with the isothermal part marked by a thick blue line. The (101) peak of tetragonal HfO_2_ is still retained at room temperature at the end of the annealing experiment.

The high-temperature stability of the 3-layer-system was tested at 300 °C for 20 h. The isothermal part of the annealing experiment is represented as a waterfall plot in Supplementary Fig.2. Three peaks are visible in the scan range from 26° to 42°, the (-111) peak from the monoclinic HfO_2_ phase at 28.1°, (101) peak from the tetragonal HfO_2_ phase at 30.3° and the (110) peak from the body centered cubic (bcc) phase of α-W at 40°. At the end of the 20 h annealing experiment, a diffractogram recorded at room temperature (brown color Supplementary Fig.2) shows the (101) peak from the tetragonal phase at 30.5° is retained in the 3-layer-system. The slight change in peak position is due to thermal expansion. It is also clearly visible that the integrated intensity of the (110) W peak is stable, indicating no loss in W in the 3-layer-system.

**Supplementary Figure 3**. Interplanar spacing *d*_-111_ of monoclinic HfO_2_ in the 3-layer-system as of function of time during annealing up to 650 °C (blue markers) and 1520 °C (red markers). The duration of the isothermal part was about 6 h.

The interplanar spacing *d*_-111_ of monoclinic HfO_2_ before the start of the annealing experiments measured at room temperature are *d*_-111_ = 0.3167 nm (blue markers) and *d*_-111_ = 0.3165 nm (red markers). Both the 3-layer-systems follow a similar pattern, initially there is an increase in the interplanar spacing distance due to thermal expansion. Later *d*_-111_ values relax and stabilize during the isothermal stage. On cooling the 3-layer-system at the end of the isothermal stage, interplanar spacing values are closer to the equilibrium value *d*_-111_ = 0.3147 nm (PDF card 00-034-0104) of bulk monoclinic HfO_2_ at room temperature. The changes of the d-values for the sample after annealing at 1520 °C, *d*_-111_ = 0.3142 nm are more pronounced in comparison to the one after 650 °C, *d*_-111_ = 0.3149 nm. The structural stability of the monoclinic HfO_2_ phase is overall sufficient for a refractory dielectric layer in metamaterial.

**Supplementary Figure 4.** Grain size values of (-111) monoclinic HfO_2_ in the 3-layer-systems annealed for 6 h at 650 °C (blue markers) and 1520 °C (red markers).

The grain size information of monoclinic HfO_2_ during heating ramp and isothermal stages at 650 °C (blue markers) and 1520 °C (red markers) are shown. The average grain size value before annealing is about 18 nm. During annealing at 650 °C, the grain size gradually increases over the time of 6 h and reaches a maximum value of 22 nm. Whereas the sample annealed at 1520 °C for 6 h, two regimes of grain growth are observed. During the ramp part of the annealing, we see a distinct increase in the grain sizes of the monoclinic phase by a factor of two, finally reaching a value up to 40 nm. The temperature dependent process of grain growth is driven by the annealing temperature, but reaches a plateau during the isothermal part of the experiment. During the 6 h at 1520°C, the HfO_2_ grains grow further but only slightly to a maximum value of about 45 nm.

**Supplementary Figure 5.**  Grain size of (110) bcc W in the 3-layer-systems annealed for 6 h at 650 °C (blue markers) and 1520 °C (red markers).

The grain size value of bcc W in the 3-layer-system during the heating ramp and isothermal stage at 650 °C (blue markers) and 1520 °C (red markers) are calculated using the Scherrer formula. The average grain size of the 3-layer-system before annealing is 14 nm. Gradual grain growth is observed for both the annealed 3-layer-system during the ramp and later stabilizes near the whole layer thickness of W during the isothermal part of the annealing experiment. The grain size at the end of the annealing experiment for the sample annealed at 650 °C is 21 nm and 1520 °C is 23 nm.

**Supplementary Figure 6.** XRD diffractogram of the vaporized constituents collected on a sapphire substrate in the chamber during the 40 h annealing experiment.

During the 40 h annealing experiment of the 3-layer-system, an additional “witness” sapphire substrate was placed on the wall of the heating chamber adjacent to the vacuum pump exit. It is observed during the isothermal stage of annealing that all the W present in the 3-layer-system is oxidized and sublimate at 1520°C. The WO_x_ that leave the 3-layer-system got redeposited on a witness sapphire substrate. The chamber wall is circulated by cooling water and therefore the sapphire substrate is comparatively at a very low temperature compared to the 3-layer-system. The distance between both is approximately 10 mm. A diffractogram of the witness sample shows presence of WO_2_ and small quantities of WO_2.7_ and W. A quantitative phase analysis results in a ratio of 90%: 5%: 5%. A reduction of the volatile WO_2_ and WO_2.7_ to W is also observed by Lou et al.^1^ in their nanostructure fabrication of WO_x_ by hot filament chemical vapor deposition.

**Supplementary Figure 7.** RBS spectra of the 3-layer-system as-prepared (green markers), annealed at 650°C (blue markers), and 1520°C (red markers).

The various elements in our 3-layer-systems HfO_2_/W/HfO_2_ on Al_2_O_3_ substrates were investigated by means of Rutherford Backscattering (RBS) after preparation and after annealing at 650°C (blue markers) and 1520°C (red markers). All samples were covered with Au and Pt for latter TEM investigations. For the chemical analysis, a 2 MeV He-ion beam with a beam current of 20 nA and a detector backscattering angle of 160° was employed. The sensitivity of depth profiling was improved by changing the incidence angle from 7° to 45° (not shown here) in order to determine if the signals originate from surface or underlying layers. At high energies, right side of the figure, the heavy elements are well separated. First, the Au/Pt signal from the additional layers for TEM preparation. Then, the Hf edge from the top layer, W from the second layer and again Hf from the third layer are clearly visible. After a large gap of no signal, the signals of the light elements like Al and O are detected at low energies. It is important to notice the sharp edge at channel 385 for the as prepared sample that indicates the presence of W in the film. There is no modification due to annealing at 650 °C after 6 h. But, after 1520 °C, the yield of W is strongly reduced from 1.0 e17 atoms cm^-2^ to 0.23 e17 atoms cm^-2^, which confirms the strong loss of W. About 75% of the HfO_2_-sandwiched W layer is gone. Additionally, the stoichiometry of HfO_2_ was estimated to be slightly over-stochiometric (HfO_2.4_) for the sample annealed at 1520 °C using simulations. This result is of importance, as it validates that W does not get the O_2_ from HfO_2_ layer, but from the residual oxygen in the annealing chamber.

**Supplementary Figure 8.** SEM cross section of the 3-layer-system annealed at different temperatures for a period of 20 h. a) 300 °C, b) 650°C, c) 800 °C, d) 1000 °C, e)1240°C and f) 1520 °C. All the images have the same scale as indicated in image (a).

In the Supplementary Fig.8 a & b, the HfO_2_ layers do not contain any void and the W layer looks uniform. At temperatures above the tetragonal-to-monoclinic transformation in HfO_2_ (Supplementary Fig.8 c to e), voids are present in the HfO_2_ layers and parts of W is found missing in the 3-layer-system due to sublimation. In the last image of the sequence Supplementary Fig.8 (f), no presence of W is visible in the 3-layer-system anymore. The loss of W is the main failure mechanism in W/HfO_2_-based multilayered metamaterials due to oxidation and sublimation through these transport channels in HfO_2_ layers.

REFERENCES

1. Lou, J. *et al.* The influence of filament temperature and oxygen concentration on tungsten oxide nanostructures by hot filament metal oxide deposition. *J. Phys. D. Appl. Phys.* **41**, (2008).
